# STAT5-Interacting Proteins: A Synopsis of Proteins that Regulate STAT5 Activity

**DOI:** 10.3390/biology6010020

**Published:** 2017-03-11

**Authors:** Ashley A. Able, Jasmine A. Burrell, Jacqueline M. Stephens

**Affiliations:** 1 Adipocyte Biology Laboratory, Pennington Biomedical Research Center, Baton Rouge, LA 70808, USA; Ashley.Able@pbrc.edu (A.A.A.); Jasmine.Burrell@pbrc.edu (J.A.B.); 2 Department of Biological Sciences, Louisiana State University, Baton Rouge, LA 70803, USA

**Keywords:** JAK, STATs, signaling

## Abstract

Signal Transducers and Activators of Transcription (STATs) are key components of the JAK/STAT pathway. Of the seven STATs, STAT5A and STAT5B are of particular interest for their critical roles in cellular differentiation, adipogenesis, oncogenesis, and immune function. The interactions of STAT5A and STAT5B with cytokine/hormone receptors, nuclear receptors, transcriptional regulators, proto-oncogenes, kinases, and phosphatases all contribute to modulating STAT5 activity. Among these STAT5 interacting proteins, some serve as coactivators or corepressors to regulate STAT5 transcriptional activity and some proteins can interact with STAT5 to enhance or repress STAT5 signaling. In addition, a few STAT5 interacting proteins have been identified as positive regulators of STAT5 that alter serine and tyrosine phosphorylation of STAT5 while other proteins have been identified as negative regulators of STAT5 via dephosphorylation. This review article will discuss how STAT5 activity is modulated by proteins that physically interact with STAT5.

## 1. Introduction

### 1.1. JAK-STAT Pathway

The JAK-STAT signaling pathway transmits extracellular signals to the nucleus and regulates a variety of cellular activities including apoptosis, differentiation, proliferation, and immunological responses. This pathway consists of receptor-associated Janus kinases (JAKs), signal transducers and activators of transcription (STATs), and a cytokine or hormone receptor [1]. The JAKs are a family of tyrosine kinases that are activated by the binding of ligands that includes growth factors, hormones, interferons, and a variety of cytokines to their specific receptors. Mammals have four JAKs: JAK1, JAK2, JAK3, and Tyrosine kinase 2 (TYK2) [2].

**Figure 1:**
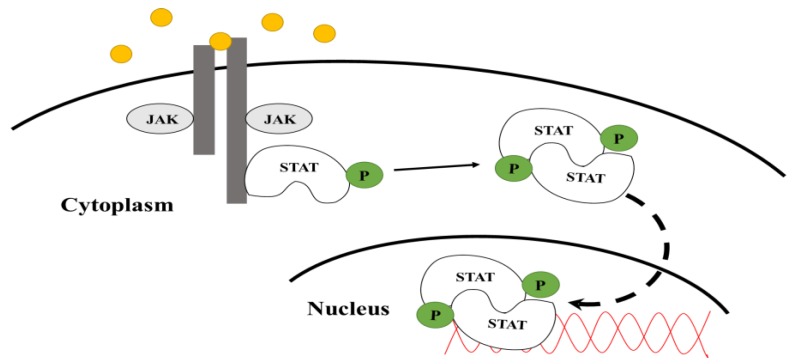
JAK-STAT signaling pathway. Upon phosphorylation of the receptor and JAK kinase, a STAT protein associates with the receptor where it is activated via tyrosine phosphorylation by JAK. Upon phosphorylation, active STATs form a dimer and translocate to the nucleus where they modulate transcription.

Upon ligand binding to the receptor, JAKs tyrosine-phosphorylate themselves as well as the receptor [3], creating a binding site for the SH2 (Src Homology 2) domain containing STAT proteins. The STATs are then activated via tyrosine phosphorylation by JAKs [3,4]. As shown in Figure 1, the activated STATs undergo dimerization and translocate to the nucleus of the cell where they bind to specific promoter regions of DNA to regulate the transcription of target genes [4]. The JAK-STAT pathway is regulated by positive and negative effectors. Activators include signal-transducing adapter molecules (STAMs) and proteins containing SH2 domains [4], while inhibitors include suppressors of cytokine signaling (SOCS) and tyrosine phosphatases [5]. STAT interacting proteins have been shown to act as both activators and inhibitors. 

### 1.2. Signal Transducers and Activators of Transcription (STATs)

**Figure 2:**
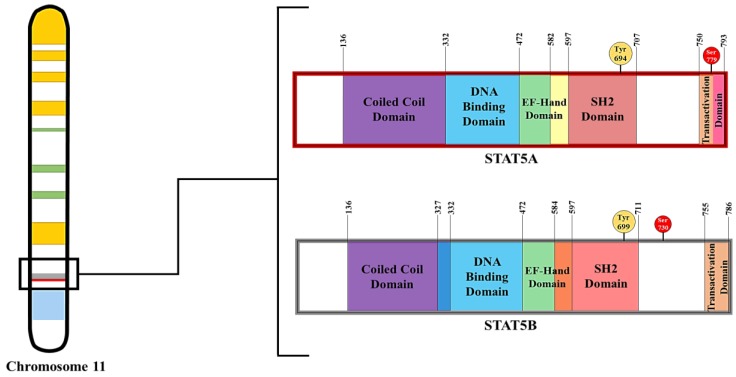
Structural domains and phosphorylation sites of STAT5 as found on Chromosome 11 in Mus musculus species. STAT5 proteins are composed of several domains as indicated in the figure. Tyrosine phosphorylation in the SH2 domain (shown in yellow) are required for STAT5 activation. Serine phosphorylation (shown in red) has been shown to modulate STAT5 activity.

Signal Transducers and Activators of Transcription (STATs) are the primary substrates for JAKs and they mediate the effects of cytokines, growth factors, several hormones, and various pathogens in mammals [6]. There have been seven STAT proteins discovered in mammals. There are seven STAT proteins: STATs 1, 2, 3, 4, 5A, 5B, and 6, all of which consist of a helical N-terminal domain (ND), a coiled coil (CC) domain, a DNA-binding domain (DBD), a helical linker (LK), a Src homology 2 (SH2) domain, and a transactivation domain (TAD) located in the C-terminus region [6]. The SH2 domain is essential for the recruitment of the STAT proteins to the hormone/cytokine receptor. Under basal conditions, inactive STATs are located in the cytosol [7]. Once they are recruited to the activated JAK/receptor complex and tyrosine-phosphorylated within the SH2 domain (refer to Figure 2) by JAKs, they form dimers and/or tetramers, translocate to the nucleus, and associate with promoter regions such as Gamma Activated Sequence (GAS) elements. The binding affinity of individual STATs to specific sites varies [6], and the interaction of STATs with gene promoters can inhibit or enhance the expression of its target genes [8].

### 1.3. STAT5 Characteristics and Functions

In mammals, STAT5 proteins have been associated with many functions including cell differentiation [7,9,10], lipid mobilization [11], and lymphocyte development [12]. STAT5 refers to two proteins: STAT5A and STAT5B, which share 94% structural homology, but are transcribed from separate genes. As shown in Figure 2, STAT5A consists of 793 amino acids while STAT5B consists of 786 amino acids in murine species [13]. STAT5A and STAT5B have molecular weights of 94 and 92 kD [14], respectively. The most significant difference between STAT5A and STAT5B is 20 amino acids in the transactivation domain (TAD) of STAT5A, as denoted by the pink bar in Figure 2. Both STAT5A and STAT5B are activated by phosphorylation at Tyr694 and Tyr699, respectively, by JAK2, which is activated by the binding of numerous cytokines and hormones including growth hormone (GH), erythropoietin (EPO), prolactin (PRL), and several interleukins (ILs) to their receptors [13]. JAK2 and STAT5 are important for IL-3 and GM-CSF (granulocyte macrophage colony-stimulating factor)-regulated macrophage function [15]. Like other STATs, STAT5 activity is regulated via inhibitory mechanisms, such as SHP-1 phosphatase dephosphorylation and negative feedback loops involving CIS (cytokine-inducible SH2-containing protein) and SOCS (suppressor of cytokine signaling) proteins [16]. Covalent modification by acetylation and deacetylation can also modulate STAT5 activity (reviewed in [17]).

STAT5A has been found to be more prevalent in mammary tissue while STAT5B expression is more enriched in muscle and liver [13]. Studies performed in mice revealed that a deficiency of STAT5A results in the impairment of mammary glands and cellular differentiation during pregnancies, while the loss of STAT5B prominently affects growth and sexual dimorphism [13,18]. STAT5A-deficient mice exhibit decreased expression of a bcl-2 like gene called A1 that results in the suppression of cell growth [14]. There is some evidence of compensatory signaling of STAT5B in STAT5A-deficient mice [19]. In addition to these functions, STAT5 has prominent roles in immune function [10,20], mammary development and function [14,19,21,22], and oncogenesis [23,24]. In the last two decades, several laboratories have identified the importance of STAT5A in adipocyte development in vitro and in vivo [7,25,26,27,28].

### 1.4. Tools for Understanding the Function of STAT5 Proteins

Since STAT5 proteins are expressed in many tissues and have various functions, a variety of tools have been used to study the role of these multifunctional proteins. As indicated above, knockout models have been generated to study the function of STAT5. Ectopic expression studies in vitro and in vivo have also been used to understand STAT5 function. In addition, site-specific mutagenesis has been employed to determine the role of specific STAT5 covalent modifications. However, one approach that has not received a lot of attention is the identification of novel STAT5 interacting proteins. In the past two decades, several proteins have been shown to physically associate with STAT5. These studies have provided insight into the specific functions of STAT5. This review will focus on those proteins and their roles in modulating STAT5 function.

## 2. STAT5 Interacting Proteins

### 2.1. Interactions with Cytokine/Hormone Receptors

STAT5 was originally named mammary gland factor (MGF) because it was initially discovered in mammary gland cells and shown to regulate the expression of β-casein [29], an abundant milk protein. STAT5, like all STATs, bind to receptors to be activated by JAKs (refer to Figure 1). Hormones that activate STAT5 mediate their biological actions via receptors that physically associate with STAT5 proteins. The tyrosine phosphorylated receptors that STAT5 has been shown to interact with include prolactin receptor (PRL), growth hormone receptor (GHR), IL-3R (interleukin 3 receptor), and EPOR (erythropoietin receptor) [30,31,32].

### 2.2. Transcriptional Modulators

#### 2.2.1. Proteins That Enhance STAT5 Transcriptional Activity

**Figure 3:**
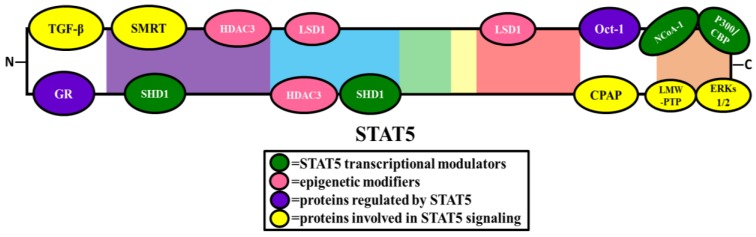
Binding sites of proteins that physically interact within the STAT5 domain. Various interacting proteins have been shown to transcriptionally modulate STAT5 (green proteins), act as epigenetic modifiers (pink proteins), can be modulated by STAT5 (purple proteins), or are involved in STAT5 signaling (yellow proteins).

**Figure 4:**
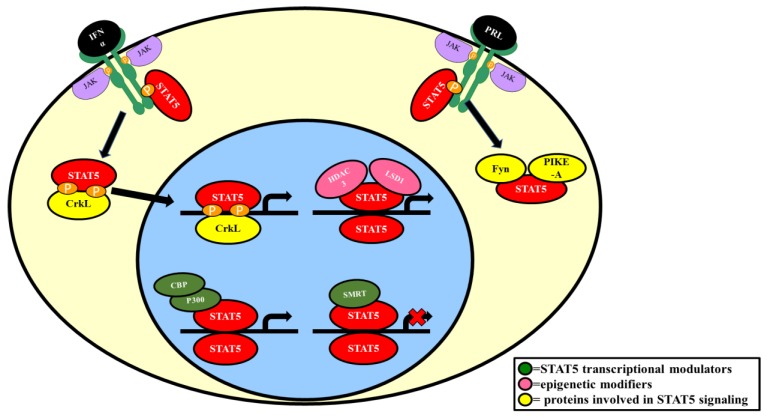
Protein interactions that modulate STAT5 activity. Schematic diagram depicting the regulation of STAT5 signaling and transcriptional activity.

Like other transcription factors, STAT5 interacts with proteins involved in the general transcription factor machinery. CBP (CREB-binding protein) and p300 are nuclear coactivators that exhibit histone acetyltransferase activity and have been shown to play a role in the transcriptional activation of both STAT5A and STAT5B [33]. In vitro studies, using yeast two hybrid assay and co-immunoprecipitation experiments with COS7 cells, have shown that p300 physically interacts with STAT5 in a prolactin-induced manner. As illustrated in Figure 3, there is evidence that p300/CBP binds the carboxy-terminal transactivation domain of STAT5 and that p300 is responsible for enhancing STAT5A and STAT5B transcriptional activity (refer to Figure 4). Reporter assays using HeLa cells transfected with the β-casein promoter-luciferase construct and ectopically expressing STAT5 and p300 showed that p300 increased prolactin-induced STAT5 transcriptional activity [33].

NcoA-1 (nuclear receptor coactivator 1), also known as SRC-1 (steroid receptor coactivator 1), is a nuclear coactivator known to coactivate various nuclear receptors such as PR, GR, ERα, TR, RXR, HNF4α, and PPARγ [34]. NcoA-1 has also been shown to physically interact with the coactivator complex, p300/CBP [35], and there is evidence that NcoA-1 physically interacts with STAT5. Co-immunoprecipitation experiments in 293T cells transfected with GFP-STAT5A and NCoA-1 confirmed the interaction [36]. When the amino acids within the alpha-helical region of the transactivation domain at positions 751–753 were deleted or mutated, NCoA-1 was no longer able to interact with STAT5A [36], indicating that the alpha-helical region within the TAD (Transactivation domain) is necessary for NcoA-1 binding to STAT5A (refer to Figure 3). To determine the function of this interaction, ChIP (chromatin immunoprecipitation) assays were performed in prolactin-stimulated HC11 cells and demonstrated that the STAT5A/NCoA-1 complex binds to a STAT5 binding site in the Cish (cytokine inducible SH2 containing protein) promoter [36].

#### 2.2.2. Proteins that Repress STAT5 Transcriptional Activity

SMRT (silencing mediator for retinoic acid receptor and thyroid hormone receptor) is a corepressor for various members of the nuclear receptor family. Although STATs are not members of the nuclear receptor superfamily, SMRT was found to interact with both STAT5A and STAT5B in a yeast two-hybrid screen [37]. In vitro protein binding assays, as well as immunoprecipitation experiments in native and transfected cells have confirmed that SMRT physically interacts with both STAT5A and STAT5B, and additional yeast two-hybrid experiments using STAT5 mutants identified the N-terminal coiled-coil region of STAT5A and STAT5B as the critical site for this association [37] (refer to Figure 3), which has been shown to repress STAT5 transcription. Overexpression of SMRT in 32D cells resulted in a down regulation of two STAT5 target genes; oncostatin M (OSM) and cytokine-inducible SH2 containing protein (Cish) [37].

SHD1 (Sac3 domain-containing protein) is a protein that shares homology with the yeast protein, Sac3 (suppressor of actin 3). SHD-1 has been shown to have a role in mitotic progression [38]. Co-immunoprecipitation experiments using nuclear extracts from BaF3 cells showed that endogenous SHD1 interacts with STAT5 [38]. Yeast two-hybrid assays using deletion mutants of STAT5B determined that SHD1 binds to the SH2 domain or the coiled-coil domain [38] (refer to Figure 3). SHD1 was also shown to be induced by various cytokines and hormones in a dose-dependent manner, suggesting a potential role in modulating STAT5 transcriptional activity [38]. Furthermore, reporter assays using 293T cells transfected with the β-casein promoter-luciferase construct and ectopically expressing SHD1 or STAT5 showed that SHD1 represses STAT5 transcriptional activity [38].

SOCS proteins can bind directly to tyrosine kinases to deactivate them, but can also block docking sites on cytokine receptors to inhibit the activation of STATs in the JAK/STAT Pathway [39]. While the SOCS family was discovered over a decade ago, only SOCS1 through 3 have been substantially researched. Recent evidence has shown that SOCS7 interacts directly with STAT5 and inhibits its activity [40]. In these experiments, SOCS7 inhibited the phosphorylation of both STAT3 and STAT5 by prolactin (PRL) or leptin [40]. Moreover, the expression of SOCS-7 in leukocytes impaired PRL and GH signaling and inhibited STAT5 activation [40].

#### 2.2.3. Role of STAT5 in Oct-1 Transcriptional Activity

Cyclin D1 is involved in regulation of the cell cycle and is a STAT5 target gene [41]. There is evidence from cyclin D1-luciferase reporter assays that STAT5 binds at -481 bp of the cyclin D1 promoter [41]. Oct-1 has also been shown to have a role in the transcriptional regulation of cyclin D1. Oct-1 (Octamer-Binding Protein 1) is a transcription factor ubiquitously expressed in the nucleus that contains a POU (pituitary specific, octomer transcription factor, Unc-86) domain, a DNA binding domain that recognizes the octamer motif [42]. Co-immunoprecipitation experiments using nuclear extracts from thrombopoietin stimulated UT7-mpl cells have shown that Oct-1 physically interacts with the carboxy-terminal of STAT5A in the nucleus [42] (refer to Figure 3), and that this association is necessary for activating the cyclin D1 promoter and regulating cycling D1 expression [42]. 

### 2.3. Association of STAT5 and Members of the Nuclear Receptor Family

#### 2.3.1. Association with PR

PR (progesterone receptor) is a nuclear receptor that has been widely investigated in the mammary gland and has a critical role in mammary gland growth and differentiation [43,44,45]. Independent co-immunoprecipitation experiments have determined that PR physically interacts with STAT5A in the nucleus of HeLa cells, C4-HI tumors, and T47D cells [46,47]. Experiments using progestin-stimulated primary mouse mammary epithelial cells (MECs) from STAT5A knockout mice have established that STAT5A functions as a coactivator in the regulation of the three PR target genes; receptor activator of nuclear factor kappa-B ligand (RANKL), wingless-type MMTV integration site family member 4 (Wnt4), and amphiregulin (Areg) [48].

In addition to PR, FGFR-2 (Fibroblast growth factor receptor 2) has also been shown to have a fundamental role in mammary gland development [49]. FGFR-2 is typically a receptor tyrosine kinase, but can translocate to the nucleus [50,51]. Co-immunoprecipitation experiments demonstrated that FGFR-2 physically interacts with STAT5A within the nucleus of C4-HI tumors and T47D cells [47], and this association has been proposed to be involved in breast cancer [47].

#### 2.3.2. Association with GR

**Figure 5:**
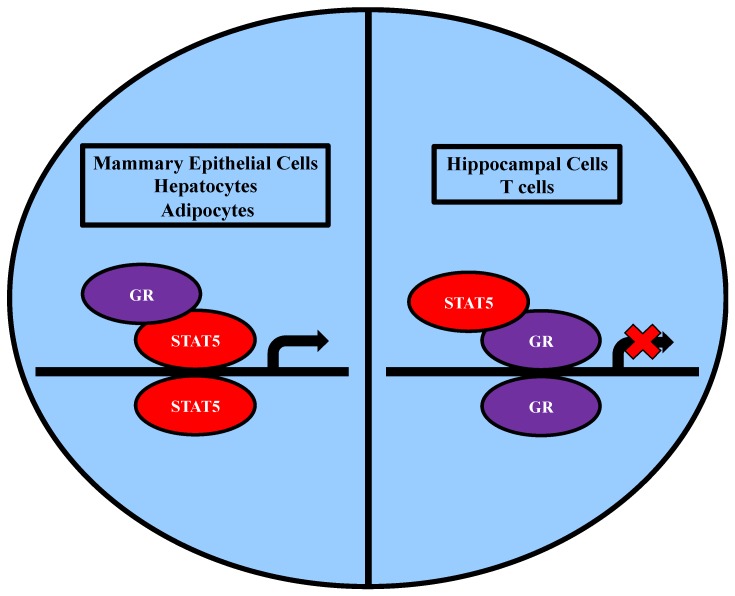
STAT5 and GR bind DNA to regulate gene expression. In mammary epithelial cells, hepatocytes, and adipocytes, there is evidence that GR acts as a coactivator in STAT5 transcriptional activity. In hippocampal cells and T cells, there is evidence that STAT5 acts as a corepressor of GR transcriptional activity.

GR (glucocorticoid receptor) is a steroid receptor found in all cell types that mediates the actions of cortisol and other glucocorticoids and has cell-specific functions. Upon ligand binding, GR dimerizes and translocates to the nucleus. GR has been shown to physically interact with both STAT5A and STAT5B in a variety of cell types including mammary gland cells, hepatocytes, pancreatic acinar cells, hippocampal cells, tumor cells, immune cells, and adipocytes [52,53,54,55,56,57]. It is well established that both STAT5A and GR have prominent roles in milk protein gene transcription within the mammary gland, so it is not surprising that most research conducted on the GR/STAT5A interaction has been performed in mammary epithelial cells. Within mammary cells, prolactin induces STAT5A/STAT5B activation and glucocorticoids induce GR activation. Co-immunoprecipitation experiments in COS7 cells co-transfected with STAT5, glucocorticoid receptor, and the prolactin receptor showed that GR physically associates with STAT5 when stimulated with prolactin and dexamethasone [52]. Additional independent co-immunoprecipitation experiments using HC11 cells have confirmed that endogenous GR and STAT5 physically interact under the same stimulating conditions throughout mammary gland development [58]. Although GR typically binds DNA at glucocorticoid response elements (GRE) to activate gene transcription, it has been shown to function differently in the regulation of β-casein gene transcription. COS7 cells were co-transfected with GR mutant variants lacking the ability to bind GREs, STAT5, prolactin receptor, and the β-casein-luciferase reporter gene, to show that the DNA-binding domain (DBD) of GR is not a requirement for its ability to enhance STAT5 transcriptional activation [59], indicating that GR acts as a coactivator of STAT5 during mammary gland development (see Figure 5).

In addition to mammary cells, the GR/STAT5B association in hepatocytes has also shown that GR can act as a coactivator in STAT5 transcriptional regulation [56]. Mice with inactive GR specifically in hepatocytes have impaired growth [60], indicating the importance of GR in hepatocytes for postnatal growth. Additional independent studies in mice lacking STAT5, or lacking both GR and STAT5 in hepatocytes showed a similar reduction in growth, which indicated that the role of GR in promoting growth is mediated by STAT5 [56]. Moreover, co-immunoprecipitation experiments using liver extracts from GH-treated mice showed that GR physically associates with STAT5B [56]. Additional independent co-immunoprecipitation experiments conducted using liver extracts from untreated or GH-treated mice with N-terminally truncated STAT5B have shown that GR physically interacts with the N-terminus of STAT5B [56] (refer to Figure 3). Expression profiles of livers from mice lacking GR in hepatocytes or from mice lacking STAT5 in hepatocytes versus their wild-type counterparts were compared and found to have similar patterns [56]. Genes whose expression was similarly altered by GR and STAT5 deletions included male-predominant genes, GH-responsive genes, steroid dehydrogenases, somatomedin mediators, and ribosomal protein genes [56]. In addition, livers from mice without the N-terminus in STAT5B and mice lacking GR in hepatocytes exhibited similar expression profiles, which showed down regulation of the STAT5-responsive genes, IGF-1 and ALS. These two genes are shown to be partially dependent on GR and involved in promoting growth and sexual maturation [56]. Together these findings indicate that GR acts as a coactivator for STAT5B transcriptional activity in the promotion of growth and sexual maturation [60,61]. STAT5 proteins also physically interact with GR in the nucleus of GH-treated 3T3-L1 adipocytes [57], suggesting that GR may act as a coactivator for STAT5 in fat cells as well as hepatocytes.

In addition to GR serving as a coactivator in STAT5 transcriptional regulation, STAT5 has also been shown to repress GR gene transcription in hippocampal cells and T cells (refer to Figure 5) [54,55]. Co-immunoprecipitation experiments using nuclear extracts from mouse hippocampal HT22 cells showed that GR physically interacts with STAT5 [55]. There is evidence that IFN-α treatment in HT22 cells results in the inhibition of DEX-induced MMTV (dexamethasone-induced mouse mammary tumor virus)-luciferase activity and the ability of GR to bind DNA [55]. STAT5 is known to be activated by IFN-α in embryonic fibroblasts [62]. Knockdown of STAT5 expression in HT22 cells prevented the inhibition of DEX-induced MMTV-luciferase activity by IFN-α [55]. This indicates that STAT5 has a role in repressing GR mediated gene transcription. Similar results have been obtained in CTLL-2 T cells treated with IL-2 [60].

### 2.4. Epigenetic Modifiers

LSD1 (lysine specific demethylase 1) and HDAC3 (histone deacetylase 3) are epigenetic modifiers that are typically associated with the modulation of histone activity. Recently, LSD1 and HDAC3 have been shown to interact with STAT5A. Immunoprecipitation experiments were conducted using nuclear extracts from IL-3 stimulated pro-B Ba/F3 cells with BirA biotinylated STAT5A [63]. The specific binding regions were determined using 35S-LSD1 and 35S-HDAC3 pull-downs with GST-STAT5A sub-fragments. LSD1 was shown to interact with the DNA-binding linker and the SH2 domain. HDAC3 was shown to interact with the DNA-binding linker, SH2 domain, and the coiled coil domain [63] (refer to Figure 3). ChIP-seq experiments have shown that STAT5A, LSD1, and HDAC3 have many overlapping binding regions within the genome [63]. RNA-seq performed in Ba/F3 cells with either STAT5A or LSD1 knocked down has revealed co-regulation among 3 genes; Il4ra, Gfi1b, and Cnr2 [63]. Based on the ChIP-seq and RNA-seq data, it appears that LSD1 and HDAC3 function to activate or repress STAT5A transcriptional activity [63] (refer to Figure 4). However, the direct effect of this interaction on STAT5A transcriptional activity has not been elucidated. Overall, the relevance for the LSD1/HDAC3/STAT5A interaction network remains unclear and additional studies are necessary to determine the function of these interactions.

EZH2 (enhancer of zeste homolog 2) is a histone-lysine N-methyltransferase enzyme that participates in the methylation of DNA and plays an important role in the regulation of transcription [64]. STAT5 has been shown to physically interact with EZH2 in HCT116 human colon cancer cells by co-immunoprecipitation experiments [65]. To determine the function of this interaction, ChIP assays were performed in HCT116 cells. These studies demonstrated that both EZH2 and STAT5 bind to the C9orf140 gene [65]. EZH2 and STAT5 were independently knocked down and showed decreased levels of C9orf140, suggesting that the interaction of STAT5 and EZH2 plays a role in inducing transcription of C9orf140 and promoting tumorigenesis [65]. In contrast, the interaction of STAT5 and EZH2 in pro-B cells plays a role in repressing the transcription of Igk [66]. Co-immunoprecipitation and ChIP assays using IL-7 stimulated Irf4−/−Irf8−/− pre-B cells and a STAT5 specific antibody revealed that the EZH2 was only recruited to STAT5 when STAT5 was in the tetrameric form and bound to DNA [66]. This interaction of EZH2 and STAT5 appears to play a role in B lymphopoiesis. STAT5 is also known to play a role in the differentiation of mammary alveoli during pregnancy. Hence, the role of EZH2 in this process has also been examined. The loss of EZH2 from mammary stem cells resulted in enhanced differentiation of alveolar epithelium and the activation of mammary-specific STAT5 target genes during pregnancy [67]. In addition, these studies showed that EZH1 and EZH2 can serve redundant functions in the formation of mammary alveoli, but the presence of EZH2 was necessary to control progressive differentiation of milk secreting epithelium during pregnancy [67]. Overall, studies in the three different tissue types show that the methyltransferase and transcriptional coactivator EZH2 can modulate the several activities of STAT5.

### 2.5. Proteins Involved in STAT5 Signaling

#### 2.5.1. Proteins That Modulate STAT5 Phosphorylation

The tyrosine phosphorylation of STAT5 proteins results in their nuclear translocation and ability to modulate transcription [68]. The requirement of the tyrosine phosphorylation for STAT5 nuclear translocation and transcriptional activity was shown by site-directed mutagenesis of tyrosine694 and expression in COS cells [68]. In addition to tyrosine phosphorylation, STAT5 proteins have been shown to be regulated by serine phosphorylation. The role of this serine phosphorylation is less clear, but it appears to modulate DNA binding affinity [69] and contribute to the transcriptional activity of both STAT5A and STAT5B in a promoter-dependent manner [70]. STAT5 can be serine-phosphorylated by MAPK. ERKs (extracellular regulated kinases) are ubiquitous serine/threonine kinases belonging to the MAPK (mitogen-activated protein kinases) family. ERKs 1/2 have been shown to interact with STAT5A under basal and growth hormone (GH)-stimulated conditions [71]. This physical interaction has been examined in vitro using co-immunoprecipitation experiments with extracts from CHO cells transfected with rat GH receptor. Upon GH treatment, ERKs 1 and 2 are activated by phosphorylation and have been shown to phosphorylate STAT5A on serine780 within the C-terminal transactivation domain (TAD).

The physical interaction between ERK1 and STAT5A appears to have a role in the modulation of STAT5A activity [71]. Studies using COS cells transfected with SPI-GLE1-luc, a STAT5-reporter luciferase construct containing the serine protease inhibitor GAS-like element 1, and ectopically expressing GHR, STAT5A, wild-type ERK, or inactive kinase ERK1 have demonstrated that ERK1 kinase activity is necessary for GH-induced reporter gene expression [71]. Currently, the biological importance of serine phosphorylation of STAT5 proteins remains to be elucidated, but appears to play a modest role in transcriptional activation and DNA binding. Serine725 in STAT5A and serine730 in STAT5B have been shown to be phosphorylated upon prolactin or interleukin-2 stimulation [72]. When serine725 and serine730 were mutated and expressed in COS-7 cells, there was no effect on transcriptional activation of a prolactin-responsive promoter or the ability of STAT5A and STAT5B to bind DNA [72]. However, independent experiments using GH stimulated COS-1 and HepG2 cells demonstrated that phosphorylation of serine725 in STAT5A at and serine730 in STAT5B was required in order to achieve full activation of a promoter highly active in liver cells [70].

#### 2.5.2. Proteins That Modulate STAT5 Dephosphorylation

Protein tyrosine phosphatases (PTPs) have been shown to have a fundamental role in regulating JAK/STAT signaling [73,74]. In IL-2 stimulated CTLL-20 cells, the cytosolic PTP, SHP-2 (SH2 domain containing protein tyrosine phosphatase), was shown to dephosphorylate STAT5A on tyrosine694 and STAT5B on tyrosine699 [75]. This modification occurred in the cytosol and was not dependent on STAT nuclear translocation. These results suggest that SHP-2 functions to negatively regulate STAT5 activity [75].

Dual-specificity phosphatases (DUSPs) are a family of type-I cysteine-based protein tyrosine phosphatases that act on both tyrosine and serine/threonine residues [76,77] and are characterized by the dephosphorylation of two residues on a substrate. One member of this family, DUSP4, is of interest for its ability to interact with STAT5B [78,79]. DUSP4, also referred to as a mitogen-activated protein kinase phosphatase 2 (MKP-2), has been found to play a role in tumor suppression [76,80], regulation of mitogen-activated protein kinases (MAPKs) [81,82], and cellular signal transduction from the cell surface to the nucleus [83]. DUSP4-deficient mice were found to have increased CD4 T cell proliferation. DUSP4 negatively regulates CD4 T cell proliferation through its ability to regulate STAT5 phosphorylation [78]. In another study, the overexpression of DUSP4 resulted in the suppression of transcription factors present in a STAT5 protein complex [79]. Presumably, DUSP4 downregulates STAT5 phosphorylation and, in turn, negatively regulates its transcriptional activity. Additional studies are required to further understand the mechanism of this interaction.

Low molecular weight PTPs (LMW-PTPs) are also of interest for their interactions with STAT5. LMW-PTPs are phosphatases that play a role in controlling cell proliferation via the dephosphorylation of tyrosine kinase receptors and docking proteins [84]. LMW-PTP has also been studied for its role in oncogenesis and has been found to be a positive regulator of the onset of tumor development and growth [84]. LMW-PTP has been found to interact directly with STAT5. LMW-PTP dephosphorylates STAT5 to negatively regulate its activity [85]. Studies have revealed that LMW-PTP interacts with a sequence of nine amino acids (749–757) ending with the threonine residue at 757 in the C-terminal region of the STAT5 gene (refer to Figure 3) [85,86]. This sequence was found to be essential for the interaction and association of LMW-PTP with STAT5 [85].

#### 2.5.3. Proteins That Enhance STAT5 Signaling

CPAP (centrosomal P4.1 associated protein) is a cytosolic protein that is normally associated with centrosomes. CPAP has been shown to physically interact with the unphosphorylated and phosphorylated forms of STAT5A in 293T cells transfected with myc-tagged STAT5A and flag-tagged CPAP [87]. As shown in Figure 3, CPAP interacts with the C-terminal of STAT5A in the cytosol, and the complex translocates to the nucleus following activation by prolactin [87]. CPAP acts similarly to STAT5A in that it is phosphorylated prior to translocation. However, the effect of CPAP phosphorylation on its activity is still not known. CPAP has also been shown to associate with the STAT5A DNA-protein complex in 293T cells in response to prolactin stimulation [87]. The STAT5A target genes influenced by CPAP’s association have yet to be determined. There is also evidence that STAT5B interacts with CPAP, but this interaction occurs with much lower affinity and its function is unknown [87].

Fyn is a proto-oncogene belonging to the Src family of non-receptor tyrosine kinases. Like STAT5A, Fyn has been shown to be involved in adipogenesis. Fyn expression increases during adipocyte development and knockdown of Fyn in 3T3-L1 cells inhibits adipogenesis as shown by decreased lipid accumulation and decreased expression of adipogenic markers [28]. Co-immunoprecipitation experiments using HEK293 cells co-transfected with GFP-tagged STAT5A and HA-tagged Fyn demonstrated that Fyn physically interacts with STAT5A (refer to Figure 4). It has been proposed that Fyn stimulates STAT5A indirectly through JAK2 [28].

In addition to Fyn, PIKE-A (Phosphoinositide 3 kinase enhancer A) has also been shown to have a role in adipogenesis. Fyn phosphorylates PIKE-A on tyrosine682 and tyrosine774 [88]. PIKE-A is a GTPase that has also been shown to physically interact with STAT5A in co-immunoprecipitation experiments using HEK293 cells co-transfected with GST-tagged PIKE-A and GFP-tagged STAT5A [28]. An independent study using prolactin-stimulated HEK293 cells co-transfected with GFP-PIKE-A and various deletion mutants of myc-STAT5A showed that PIKE-A binds to the C-terminal of STAT5A in the cytosol [89] (refer to Figure 3 and Figure 4). To examine if Fyn had a role in this association, two HA-tagged Fyn mutants (constitutively active Fyn and kinase-dead Fyn) were transfected and overexpressed in HEK293 cells. Overexpression of constitutively active Fyn resulted in an increased PIKE-A/STAT5A interaction whereas overexpression of kinase-dead Fyn resulted in a decreased PIKE-A/STAT5A interaction [28]. These observations indicate that Fyn positively regulates the PIKE-A/STAT5A association. Moreover, the Fyn/PIKE-A/STAT5A interaction network is thought to play a role in adipogenesis [28].

CrkL (CT10 regulator of kinase-like proto-oncogene) is an adaptor protein responsible for linking proteins within various signaling cascades [10,90]. Like STAT5, it contains an SH2 domain, can be tyrosine phosphorylated, and can translocate to the nucleus. STAT5 can be activated by various hormones and cytokines, including IFNα, IFNβ, IL-3, GM-CSF, thrombopoietin, and erythropoietin [10,62,91,92]. Interestingly, CrkL has been shown to form a complex with STAT5 under these conditions as well. There is evidence that the SH2 domain of CrkL binds the phosphorylated form of STAT5 and that this complex can translocate to the nucleus and bind DNA to regulate gene expression [61] (refer to Figure 5). This mechanism was demonstrated using genomic DNA affinity chromatography in human lymphoma Daudi cells treated with either IFNα or IFNβ to show that STAT5 forms a DNA-binding complex with CrkL that binds specific GAS (Gamma Activated Sequence) elements in the promoters of IFN-stimulated genes [61]. In independent co-immunoprecipitation experiments using TF-1 cells, CrkL was found to physically associate with STAT5 only when stimulated with GM-CSF or IL-3 [93]. Similarly, in co-immunoprecipitation experiments using UT7/EPO cells or UT7/TPO cells, CrkL was only found to associate with STAT5 in UT7/EPO cells or UT7/TPO cells only when stimulated with erythropoietin or thrombopoietin, respectively [93]. Together, these experiments indicate the importance of the CrkL/STAT5 association in regulating gene expression for different signaling pathways.

In addition to the Ras/MAPK pathway, STAT5 is also affected by the PI3K/Akt pathway. STAT5 can physically interact with p85, the regulatory subunit of PI3K [94]. Co-immunoprecipitation experiments using Ba/F3 cells and IL-3 treatment demonstrated that p85 interacts with both unphosphorylated and phosphorylated STAT5 [94]. A similar approach was performed to demonstrate that Gab2 can physically associate with STAT5. Gab2 is a Grb2 (growth-factor-receptor-bound protein 2)-associated binder-2 and is also involved in the PI3K/Akt pathway. It serves as a scaffolding adaptor protein and becomes activated upon tyrosine phosphorylation [95]. Co-immunoprecipitation experiments using Ba/F3 and IL-3 stimulated cells have shown that Gab2 only interacts with tyrosine phosphorylated STAT5 [95]. When Ba/F3 cells were transfected with constitutively phosphorylated STAT5 and either Gab2 or mutant Gab2-3YF, the Gab2 mutant prevented cell growth and induced apoptosis. These studies revealed that the Gab2/STAT5 interaction can play a role in cell growth and survival in Ba/F3 cells [95].

#### 2.5.4. Proteins That Repress STAT5 Signaling

TGFβ (transforming growth factor) is a cytokine that plays a role in cell growth, proliferation, differentiation, and death. Immunoprecipitation experiments using cell lysates from wild-type STAT5 MEFs (mouse embryo fibroblast) have shown that TGF-β physically interacts with STAT5A. Immunofluorescent staining indicates that this interaction occurs in the cytosol, and there is evidence that TGFβ interacts with the STAT5 N-terminal domain [96] (refer to Figure 3). TGFβ expression was elevated in STAT5-null MEFs compared to wildtype MEFs, indicating a role for STAT5 in regulating TGFβ levels. In another experiment using MEFs infected with a retrovirus expressing a GFP-tagged TGFβ, GH-induced STAT5A phosphorylation was only detected in the absence of TGFβ, suggesting that TGFβ negatively regulates STAT5A activity [96].

## 3. Conclusions

The discovery of STATs over twenty-five years ago revealed a new intracellular signaling pathway that mediated the actions of dozens of different growth factors, hormones, and cytokines. The specificity of activation and function of the seven STAT proteins in mammals is still not completely understood. However, specificity is determined, at least in part, by the receptor and the specific STAT protein. STAT5 is a unique STAT family member, as there are two STAT5 proteins that are encoded for by separate genes. STATs 5A and 5B have both redundant and non-redundant functions that include the modulation of cell differentiation, lipid mobilization, lymphocyte development, and oncogenesis. The diverse array of STAT5 functions is consistent with STAT5 proteins having the cell specific functions described in this review. The identification of STAT5 interacting proteins in a variety of cell types has provided insight into the functions of STAT5 proteins. Not surprisingly, many STAT5 interacting proteins are transcriptional regulators that either increase or decrease STAT5 transcriptional activity. Most other STAT5 interacting proteins that have been identified thus far are associated with epigenetic regulation or modulation of STAT5 phosphorylation. Given the diverse functions of STAT5 in many cell types, the identification of additional STAT5 interacting proteins will likely provide substantial insight into the functions and specificity of action of these important signaling molecules.
